# Noise and mental health: evidence, mechanisms, and consequences

**DOI:** 10.1038/s41370-024-00642-5

**Published:** 2024-01-26

**Authors:** Omar Hahad, Marin Kuntic, Sadeer Al-Kindi, Ivana Kuntic, Donya Gilan, Katja Petrowski, Andreas Daiber, Thomas Münzel

**Affiliations:** 1 Department of Cardiology—Cardiology I, University Medical Center of the Johannes Gutenberg-University Mainz, Mainz, Germany; 2 German Center for Cardiovascular Research (DZHK), partner site Rhine-Main, Mainz, Germany; 3 Cardiovascular Prevention and Wellness, DeBakey Heart and Vascular Center, Houston Methodist, Houston, TX USA; 4 Leibniz Institute for Resilience Research (LIR), Mainz, Germany; 5 Department of Psychiatry and Psychotherapy, University Medical Center of the Johannes Gutenberg-University Mainz, Mainz, Germany; 6 Medical Psychology & Medical Sociology, University Medical Center of the Johannes Gutenberg-University Mainz, Mainz, Germany

**Keywords:** Vulnerable Populations, Population Based Studies, Exposomics

## Abstract

The recognition of noise exposure as a prominent environmental determinant of public health has grown substantially. While recent years have yielded a wealth of evidence linking environmental noise exposure primarily to cardiovascular ailments, our understanding of the detrimental effects of noise on the brain and mental health outcomes remains limited. Despite being a nascent research area, an increasing body of compelling research and conclusive findings confirms that exposure to noise, particularly from sources such as traffic, can potentially impact the central nervous system. These harms of noise increase the susceptibility to mental health conditions such as depression, anxiety, suicide, and behavioral problems in children and adolescents. From a mechanistic perspective, several investigations propose direct adverse phenotypic changes in brain tissue by noise (e.g. neuroinflammation, cerebral oxidative stress), in addition to feedback signaling by remote organ damage, dysregulated immune cells, and impaired circadian rhythms, which may collectively contribute to noise-dependent impairment of mental health. This concise review linking noise exposure to mental health outcomes seeks to fill research gaps by assessing current findings from studies involving both humans and animals.

## Noise as a public health challenge and trigger of chronic non-communicable diseases

**Fig. 1:**
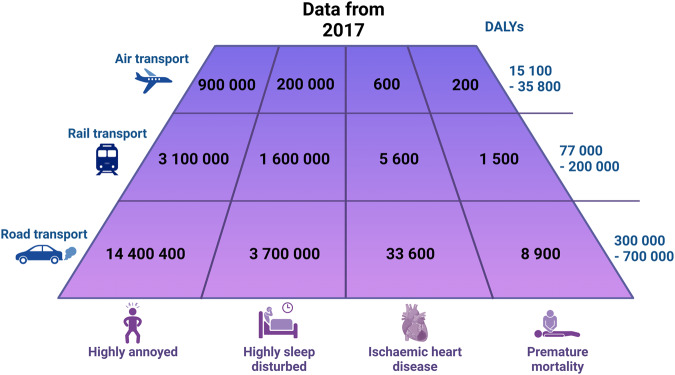
Key impacts of exposure to unhealthy noise levels, based on the Environmental Noise Directive (END) thresholds, in the European Union in 2017. One DALY equals to the loss of 1 year of healthy life attributed to morbidity, mortality, or both. The most important contributors to the total burden of disease of environmental noise are annoyance and sleep disturbance because of the large number of people affected. Adapted from [70]. DALYs disability-adjusted life years.

**Fig. 2:**
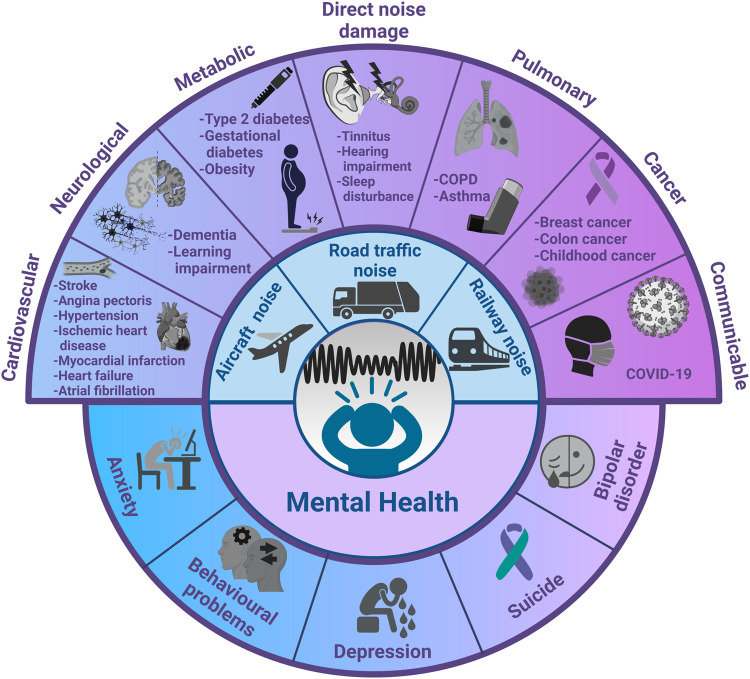
The effects of noise on different organ systems and on the mental health. Noise from different sources was previously shown to likely affect different organ systems and promote a wide variety of diseases. Detrimental effects of noise can also play a prominent role in onset and progression of many aspects of mental health, like anxiety and depression. Data derived from the following studies: [49–51, 71–87].

Noise is one of the most ubiquitous environmental pollutants, as suggested by reports from the World Health Organization (WHO) and the European Environment Agency (EEA) that noise exposure is a major public health threat affecting both physical and mental health [1, 2]. In the European Union alone, estimates indicate that at least 20% of the urban population are affected by the harmful effects of road traffic noise. Consequently, long-term transportation noise levels result in at least 18 million people being highly noise annoyed and further 5 million suffering from high sleep disturbances [2]. In addition, the WHO reported a loss of more than 1.6 million healthy life years annually due to environmental noise exposure in Western European countries [1]. Importantly, annoyance and sleep disturbance are proposed as key drivers of noise-associated non-communicable disease (NCD) onset and progression (Fig. 1) including both physical and mental health conditions [3]. Indeed, noise exposure has been implicated in a wide range of major NCDs including cardiovascular disease, metabolic disease, cancer, and respiratory disease (Fig. 2 provides an overview). We recently reviewed the cerebral consequences of environmental noise exposure in detail, suggesting that noise exposure could be an important but largely unrecognized risk factor for neuropsychiatric outcomes [4]. However, in contrast to the well-established effects of noise exposure on major NCDs, and particularly on cardiovascular disease, its effects on mental health have not been mapped in detail. This is also reflected by the omission of the quantitative details of the harms of noise on mental health consequences in reports by the WHO or the EEA. This is of concern as mental health disorders may contribute substantially to the burden of disease in the population exposed to noise. Thus, this compact review on mental health identifies some areas of future research by evaluating recent findings from human and animal studies.

## The noise/stress concept

**Fig. 3:**
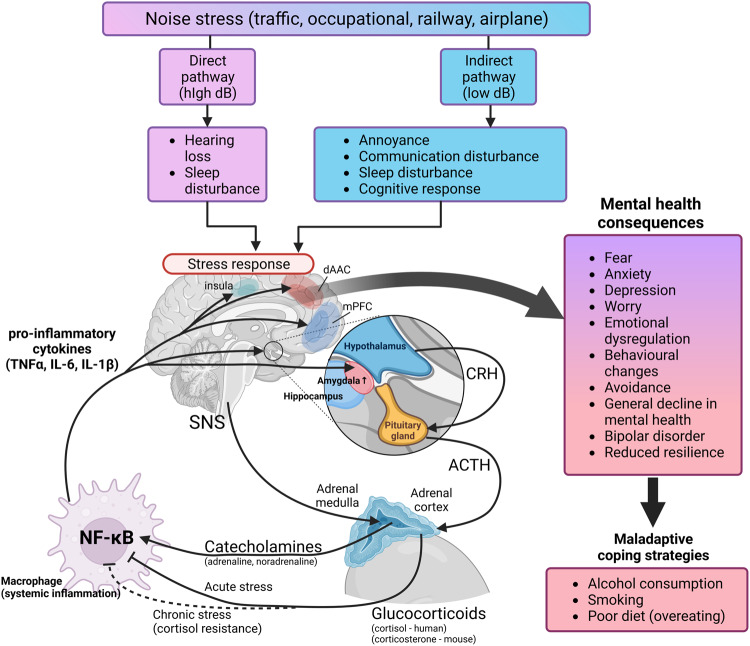
The noise/stress concept and the associated adverse mental health consequences. Noise induces the stress response through either direct (hearing loss and inner ear damage) pathway or indirect (annoyance and sleep disturbance) pathway. The stress response results in the activation of the hypothalamic–pituitary–adrenal (HPA) axis and an increase in systemic inflammation that becomes neuroinflammation, resulting in the fear and anxiety response. Prolonged exposure to a high stress response leads to maladaptive coping strategies, such as smoking or alcohol consumption. CRH (corticotropin-releasing hormone), ACTH (adrenocorticotropic hormone), NF-κB (nuclear factor kappa-light-chain-enhancer of activated B cells), SNS (sympathetic nervous system), dAAC (dorsal anterior cingulate cortex), mPFC (medial prefrontal cortex), TNFα (tumor necrosis factor alpha), IL-6/1β (interleukin 6/1β). Adapted from [27].

The association between noise exposure and adverse mental health outcomes involves a complex interplay of psychological and behavioral mechanisms. In accordance with the noise/stress concept developed by Wolfgang Babisch [5], there are two main pathways by which noise exposure causes adverse health effects. The so-called *“direct pathway”*, i.e. exposure to extreme high decibel levels (>100 dB(A)) causing direct ear organ damage, and the so-called *“indirect pathway”* related to the exposure to lower decibel levels in the range of 50–70 dB(A) that impairs daily activities, sleep, and communication. Sleep disturbance is strongly linked to mental health problems, including anxiety and depression [6]. This lower decibel noise leads to sympathetic and endocrine activation and several cognitive and emotional stress reactions, including annoyance, depressive-like states, and mental stress characterized by elevated stress hormone levels and activation of the sympathetic nervous system (Fig. 3). Noise annoyance, characterized by feelings of displeasure and discomfort, can contribute to increased stress levels and the development or exacerbation of mental health issues [3]. This noise-induced pathophysiological cascade favors not only the development and progression of mental health conditions but also of cardiovascular risk factors and cardiovascular disease [3]. Importantly, chronic mental stress per se is a well-known risk factor for both physical and mental health [7]. Even acute nighttime aircraft noise exposure induces takotsubo cardiomyopathy, also known as broken-heart syndrome, a condition triggered by emotional stress and excessive release of stress hormones [8]. In general, chronic noise annoyance/stress may impair adaptation and increase stress vulnerability, leading to decreased stress resistance and coping capacity [3]. In addition, noise exposure may promote maladaptive coping styles as indicated by recent studies demonstrating that traffic noise exposure is associated with increases in smoking, alcohol consumption, and sedentary behavior, all of which can increase the vulnerability to mental health conditions [9–11]. Learned helplessness, characterized by passive resignation due to a perceived lack of control, often arises from chronic exposure to uncontrollable stressors. These exposures trigger a sustained stress response, impacting cognitive processes and leading to a belief that a stress situation is unchangeable, which may increase the vulnerability to mental health problems. Recent research suggests an involvement of learned helplessness when it comes the adverse mental health effects of noise exposure [12].

## Mechanisms of noise-induced mental health consequences—insights from animal models

Several studies in animal models reported that environmental noise can influence inflammatory and oxidative stress pathways in the brain, leading to anxiety and depression-like behavior. A study in mice indicated that traffic noise caused hyperactivity of the hypothalamic–pituitary–adrenal (HPA) axis, leading to lower performance in all cognitive and motor tasks, a reduction of size in the hippocampal formation, medial prefrontal cortex (mPFC), and amygdala, and a reduced neuronal density in the mPFC and dentate gyrus (DG) [13]. Although the results are indicative of cognitive decline, the authors point out that the behavior of mice is suggestive of anxiety-like behavior, providing the connection to mental health decline. The same group also observed increases in anxiety-like behavior, reduced time spent exploring new object/environment even when mice were exposed to a 3000 Hz synthetic sound tone [14]. Neuroinflammation, as shown by increases in IL-1β IL-6 and TNFα in the hippocampus and prefrontal cortex, was observed in mice exposed to a synthetic noise stimulus of 80 dB [15]. These authors also observed depression-like behaviors, envisaged by a decrease in sucrose preference and reduction in times of crossings in the open-field test and the times of rearings (standing on hind legs) in the open-field test. Another study in mice showed that chronic noise exposure caused an increase in malondialdehyde (MDA) levels in the brain, together with a decrease in superoxide dismutase (SOD) and glutathione peroxidase (GPx) activity [16]. These increases in oxidative stress markers were also accompanied by greater circulating cortisol levels and impaired social interactions. A 30-day noise exposure study in rats showed that elevated plasma corticosterone levels are linked to impairment in spatial memory [17]. This was also accompanied by decreases in catalase and glutathione peroxidase activity in the medial prefrontal cortex and hippocampus, suggesting increased oxidative stress. Another study showed that plasma levels of corticosterone, adrenaline, noradrenaline, endothelin-1, nitric oxide and malondialdehyde were increased in rats chronically exposed to intermittent noise, while superoxide dismutase expression was decreased [18]. A study in spontaneously hypertensive rats showed that noise stress resulted in exaggerated glutamatergic responses in the amygdala, pointing to the activation of this important pathway [19].

Our studies in mouse models show that 4-day of exposure to aircraft noise increased levels of pro-inflammatory cytokines IL-6, inducible nitric oxide synthase (iNOS) and cluster of differentiation 68 (CD68) in mouse brains [20]. Down-regulated catalase and neuronal nitric oxide synthase (nNOS) were also observed as key factors of cerebral/neuronal damage in mice exposed to noise. These negative effects were ameliorated by the genetic deletion of the subunit of phagocytic NADPH oxidase (gp91phox), pointing to the important role of immune cell-derived oxidative stress. Interestingly, the effects were more pronounced when noise was applied during the sleeping phase of mice, which correlates well with the impairment of circadian rhythms by sleep fragmentation and deprivation [20]. Dysregulation of circadian rhythms seems to represent a hallmark of noise-induced pathomechanisms as it is clear that nighttime noise exposure is much more detrimental for humans than daytime noise [21–23]. We also observed increases in levels of circulating catecholamines (adrenaline and noradrenaline) in a mouse model of 3-day aircraft noise exposure [24]. These experimental data point to a biological state associated with anxiety- and depression-like symptoms, but more preclinical research is needed to draw a strong correlation. Mechanistic findings from animal models have been used to produce a stress response pathway that enables us to better understand the implications of noise exposure on human mental health.

## Mechanisms of noise-induced mental health consequences—stress response pathways

It is generally challenging to identify biochemical correlates of mental health, as mental health is not a single disease, but a collection of complex psychological states with overlapping signs and symptoms. However, anxiety, depression and general mental stress have been associated with activation of certain neurological and endocrine pathways. Anxiety and depression are both correlated with fear and stress via the autonomic nervous system [25]. Noise-induced stress responses activate the hypothalamic–pituitary–adrenal (HPA) axis and the sympathetic nervous system (SNS) [26]. The stress response is triggered when the hypothalamus releases corticotropin-releasing hormone (CRH) and arginine vasopressin (AVP) into the pituitary gland, further stimulating the release of adrenocorticotropic hormone (ACTH) into the circulation. ACTH then signals the adrenal cortex to release glucocorticoids and the SNS signals the adrenal medulla to release catecholamines. The overstimulation of the SNS suppresses the ability of glucocorticoids to modulate the inflammatory response, resulting in the release of pro-inflammatory cytokines [27, 28]. Likewise, chronic stress and the overproduction of glucocorticoids leads to down-regulation of their receptors in immune cells, with a subsequent loss of the ability of glucocorticoids to suppress the activation of inflammatory pathways, e.g. cytokine release, a condition called “cortisol resistance” [29]. The release of pro-inflammatory cytokines is mostly modulated by the activation of the transcription factor nuclear factor kappa-light-chain-enhancer of activated B cells (NF-κB) [30]. The inflammatory state can contribute to the maintenance of the fear and stress response by modulating the activity of the brain regions implicated in anxiety, like the amygdala, hippocampus, insula, prefrontal cortex (mPFC), and the anterior cingulate cortex (dACC) [31]. This systemic inflammatory response can in turn exacerbate neuroinflammation [32]. Pro-inflammatory cytokines, such as interleukins 1β/1α/6 (IL-1β, IL-1α, IL-6) and tumor necrosis factor alpha (TNFα), cannot penetrate the blood brain barrier, but can induce inflammatory responses in the circumventricular organs [33]. Microglia and astrocytes become activated and propagate neuroinflammation further by releasing of pro-inflammatory cytokines [34]. Activated immune cells in the brain can disrupt the blood brain barrier and lead to further influx of circulating pro-inflammatory cytokines into the brain [35].

Another important brain region associated with anxiety and depression is the amygdala [36, 37]. During conditions of external stress, the amygdala can become hyperactivated, increasing the sensitivity to environmental stimuli [38]. The increase in amygdala activity is both a source of neuroinflammation while also being susceptible to systemic inflammation [39, 40]. Oxidative stress and inflammation are almost inseparable in a diseased state, as neuroinflammation is accompanied by oxidative stress in the brain tissue [41, 42]. The release of reactive oxygen species (ROS) is a ubiquitous defense mechanism for any resident immune cells. Neuronal tissue is more susceptible to oxidative stress as neurons have membranes rich in polyunsaturated fats, making them prone to lipid oxidation [43]. In addition, dopamine, norepinephrine, and serotonin are prone to auto-oxidation, impairing synaptic signaling [44]. Nervous tissue also lacks many antioxidant defense mechanisms available to other tissues [45]. The mechanisms of noise-induced stress response are presented in Fig. 3.

## Epidemiological evidence

### Depression and anxiety

A meta-analysis by Dzhambov and Lercher reported that road traffic noise exposure was associated with 4% higher odds of depression (odds ratio (OR) 1.04, 95% CI 1.03–1.11) as well as 12% higher odds of anxiety (OR 1.12, 95% CI 1.04–1.30 both per 10 dB(A) increase in L_den_). However, it is important to acknowledge that most of the studies in the meta-analysis were cross-sectional and of lower quality [46]. In agreement, the meta-analysis by Hegewald et al. provided data supporting an association between traffic noise exposure and depression and anxiety [47]. The authors demonstrated a 12% increase in risk of depression (effect size 1.12, 95% CI 1.02–1.23 per 10 dB increase in L_den_) in response to aircraft noise exposure, while weaker risk increases of 2–3% (not statistically significant) were obtained for road traffic and railway noise exposure. A meta-analysis of nine studies indicated a 9% higher odds of anxiety (OR 1.09, 95% CI 0.97–1.23 per 10 dB increase in L_den_) due to traffic noise exposure [48]. Higher traffic noise levels were associated with depressive (OR 1.17, 95% CI 1.03–1.32) and anxiety disorders (OR 1.22, 95% CI 1.09–1.38 both per 3.21 dB increase in L_den_) in the Netherlands Study of Depression and Anxiety (*N* = 2980) [49]. A German case-controlled study investigated depression risk by aircraft, road traffic, and railway noise exposure [50]. For road traffic noise, a linear exposure-risk relationship was determined (OR 1.17, 95% CI 1.10–1.25 for L_pAeq,24h_ ≥ 70 dB vs. <40 dB). The highest risk increases were shown for aircraft noise ranging at L_pAeq,24h_ = 50–55 dB (OR of 1.23, 95% CI 1.19-1.28 for comparison < 40 dB) and for railway noise ranging at L_pAeq,24h_ = 60–65 dB (OR 1.15, 95% CI 1.08–1.22 for comparison <40 dB). Interestingly, combining all three exposures (above 50 dB L_pAeq,24h_) resulted in the most excessive risk increase of an OR of 1.42 (95% CI 1.33–1.52 with the reference group being no exposure of 40 dB or more to traffic noise of any source). In the UK Biobank, a positive association between symptoms of nerves, anxiety, tension or depression (OR 1.04, 95% CI 1.01–1.07 for ≥57.8 dB) and bipolar disorder (OR: 1.54, 95% CI 1.21–1.97 for ≥57.8 dB) and road traffic noise exposure was found, while an inverse association occurred for major depression (OR 0.95, 95% CI 0.90-1.00 for 52.1-54.9 dB) [51]. The incidence of depression due to road traffic, railway, and aircraft noise exposure (L_den_) as well as noise annoyance was examined in the Swiss cohort study on air pollution and lung and heart diseases in adults (SAPALDIA) [52]. For road traffic (RR 1.06, 95% CI 0.93–1.22) and aircraft noise exposure (RR 1.19, 95% CI 0.93–1.53 both per 10 dB L_den_) suggestive positive evidence was found for harm, while the effect of noise annoyance was more robust (RR 1.05, 95% CI 1.02–1.08 per point increase). The association between residential noise exposure during pregnancy and later depression hospitalization was examined in sample of 140,456 Canadian women [53]. Herein, strongest risk increases were found for nighttime noise exposure (hazard ratio (HR) 1.68, 95% CI 1.05–2.67 for 70 vs. 50 dB(A) L_night_). Evidence from a Korean study (*N* = 45,241) suggested self-reported exposure to occupational noise and vibration elevated the odds of anxiety in both men (OR 2.25, 95% CI 1.77–2.87) and women (OR 2.17, 95% CI 1.79–2.61 both vs. no occupational exposure to noise and vibration) [54]. Interestingly, in 2,745 subjects from the Heinz Nixdorf recall study from Germany, there was a pronounced decrease in cognitive function in response to traffic noise when comparing depressed vs. non-depressed subjects, suggesting that those with existing mental health conditions may be more vulnerable to the adverse consequences of noise exposure [55]. Suggestive evidence for an association between the use of psychotropic drugs including sleep medication, anxiolytics, and antidepressants and levels of traffic noise, noise annoyance, and sensitivity was shown by a Finnish study including 7321 subjects [56]. Results from the German Gutenberg Health Study (*N* = 11,905) indicated an association between noise annoyance due to various sources and the incidence of depression, anxiety, and sleep disturbance [57]. While data from 4508 US adolescents from an urban area indicated an association between living in a high-noise area and later bedtimes, a weaker association for depression and anxiety was found [58]. In a cohort of 2,398 men from the UK, road traffic noise exposure (OR 1.82, 95% CI 1.07–3.07 for 56–60 dB(A)), high noise annoyance (OR 2.47, 95% CI 1.00-6.13), and high noise sensitivity (OR 1.65, 95% CI 1.09-2.50) were associated with incident psychological ill-health, which was determined by a questionnaire that predominantly measures depression and anxiety [59].

### Suicide

**Fig. 4:**
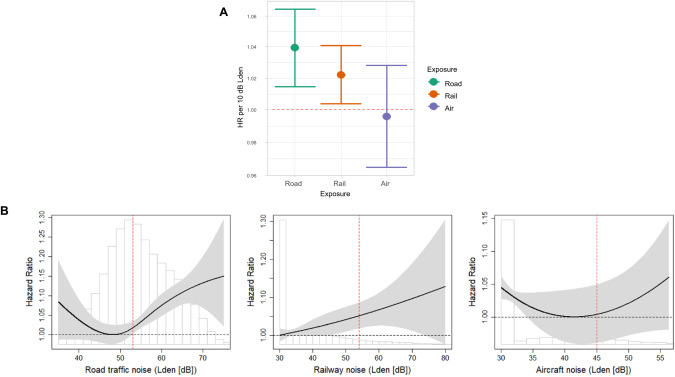
Risk of suicide and transportation noise. **A** Association (hazard ratios and 95% confidence interval) between transportation noise source (L_den_) and mortality from all intentional self-harm (ICD-10: X60–84, excl. ICD-10 ×61.8, X61.9, X81–82) after multivariable adjustment including PM_2.5_ exposure. **B** Exposure-response relationships for the association between transportation noise source (L_den_) and mortality from intentional self-harm (ICD-10: X60–84, excl. ICD-10 ×61.8, X61.9, X81–82). Vertical dashed red lines show source-specific WHO guideline levels: road traffic = 53 dB, railway = 54 dB, aircraft = 45 dB. Adapted from [60] with permission.

The Swiss National Cohort examined the association between source-specific transportation noise and suicide [60]. The authors demonstrated that road traffic and railway noise was associated with total suicides (HR 1.040, 95% CI 1.015–1.065 and HR 1.022, 95% CI 1.004–1.041, respectively per 10 dB L_den_). In contrast, this association was weaker for aircraft noise as observed risk increases starting from 50 dB were masked by an inverse association in the very low exposure range from 30 to 40 dB (Fig. 4). In the city of Madrid, short-term exposure to traffic noise was associated with emergency hospital admissions due to anxiety, dementia, and suicides [61]. Higher nighttime noise exposure was associated with elevated risks of suicide death in younger adults (HR 1.32, 95% CI 1.02–1.70), older adults (HR 1.43, 95% CI 1.01-2.02), and adults with mental illness (HR 1.55, 95% CI 1.10–2.19 all per interquartile range increase) in a Korean study (*N* = 155,492) [62].

### Behavioral problems in children and adolescents

In the Danish National Birth Cohort study (*N* = 46,940), a 10 dB increase in road traffic noise exposure from birth to 7 years of age was associated with a 7% increase (95% CI 1.00–1.14) in abnormal versus normal total difficulties scores, 5% (95% CI 1.00–1.10) and 9% (95% CI 1.03–1.18) increases in borderline and abnormal hyperactivity/inattention subscale scores, respectively, and 5% (95% CI 0.98–1.14) and 6% (95% CI 0.99–1.12) increases in abnormal conduct problem and peer relationship problem subscale scores, respectively (assessed by the parent-reported Strengths and Difficulties Questionnaire) [63]. Likewise, among schoolchildren in China, residential road traffic noise exposure was associated with increases in total/abnormal difficulties score, emotional problems, and behavioral concerns [64]. In a cohort of 886 adolescents in Switzerland aged 10–17, cross-sectionally analyzed peer relationship problems increased by 0.15 units (95% CI 0.02–0.27) per 10 dB increase in road traffic noise exposure [65]. However, this relationship was absent in longitudinal analysis. In preschool children in the city of São Paulo (*N* = 3385 children at 3 years of age and *N* = 1546 children at 6 years of age), community noise exposure above L_den_ of 70 dB and L_night_ of 60 dB was associated with impaired behavioral and cognitive development [66]. In contrast, no association was observed between prenatal or childhood road traffic or total noise exposure and emotional, aggressive, and attention-deficit/hyperactivity disorder-related symptoms in children from two European (Spain and Netherlands) birth cohorts [67]. A positive association between noise exposure at school and attention-deficit/hyperactivity disorder-related symptoms was found in a study of children aged 7–11 years in the city of Barcelona [68].

## Future research needs and conclusions

Noise exposure likely has effects on mental health since the brain represents the primary target organ of noise-mediated effects. While the effects may seem minor when examining human studies, the public health implications are significant. This is evident in reports from the WHO and the EEA, which highlight that environmental stressors such as noise have substantial and continuous impacts on large segments of the population [1, 2]. Some direct adverse phenotypic changes in brain tissue by noise (e.g. neuroinflammation, cerebral oxidative stress), feedback signaling by remote organ damage, dysregulated immune cells, and impaired circadian clock may also play important roles in noise-dependent impairment of mental health. Based on the mechanistic findings on noise research, it is evident that there is a substantial pathomechanistic overlap with mental health conditions, such as depression, that are all linked to cerebral oxidative stress and inflammation. By sharing pathomechanisms, noise can either promote the development of mental health problems or increase their severity in a bonfire fashion.

Future research needs include: preclinical noise research should deepen the mechanistic understanding of noise-induced mental health problems, allowing for drug-based interventions at different levels that target the detrimental neuronal signaling cascade. In addition, biomarkers of noise-triggered mental health harms should be identified using validated animal models in order to allow early diagnosis of vulnerable groups at higher risk of noise-inflicted mental disease. Clinical noise research should further extend the evidence base of exposure-mediated mental health effects and also investigate non-pharmacological mitigation strategies (e.g. coping mechanisms for improved resilience) such as exercise, meditation, green space availability, co-exposures, and mental health training [69]. Additional research is also needed on the benefits of technology to reduce noise (e.g noise cancellation headphones, active noise cancellation home kits, etc).

## Supplementary information

Reporting Checklist
